# Physical Properties of Peanut and Soy Protein-Based Emulsion Gels Induced by Various Coagulants

**DOI:** 10.3390/gels8020079

**Published:** 2022-01-26

**Authors:** Shaobing Zhang, Yushan Jiang, Shuyan Zhang, Lin Chen

**Affiliations:** College of Food Science and Engineering, Henan University of Technology, Zhengzhou 450001, China; 201892062@stu.haut.edu.cn (Y.J.); 18336466993@163.com (S.Z.); linchen11251216@163.com (L.C.)

**Keywords:** peanut proteins, soy proteins, conarachin, arachin, emulsion gel

## Abstract

Emulsions of peanut and soy proteins, including their major components (arachin, conarachin, glycinin and β−conglycinin), were prepared by ultrasonication (300 W, 20 min) at a constant protein concentration (4%, w/v) and oil fraction (30%, v/v). These emulsions were then induced by CaCl_2_, transglutaminase (TGase) and glucono-δ-lactone (GDL) to form emulsion gels. The optimum coagulant concentrations were obtained for peanut and soy protein-stabilized emulsion gels, such as CaCl_2_ (0.15 and 0.25 g/dL, respectively), TGase (25 U/mL) and GDL (0.3% and 0.5%, w/v, respectively). For the CaCl_2_-induced emulsion gels, the hardness of the β−conglycinin gel was the highest, whereas that of the conarachin gel was the lowest. However, when TGase and GDL were used as coagulants, the strength of the conarachin emulsion gel was the best. For the GDL-induced emulsion gels, microstructural analysis indicated that the conarachin gel showed more homogeneous and compact structures. The gelation kinetics showed that the storage modulus (G′) of all the GDL-induced emulsions increased sharply except for the arachin-stabilized emulsion. The interactive force nature varied between conarachin and arachin emulsion gels. This work reveals that peanut conarachin could be used as a good protein source to produce emulsion gels when suitable coagulants are selected.

## 1. Introduction

Emulsion gels have a certain mechanical strength filled with emulsion droplets. They can be used as fat substitutes or as delivery systems to embed bioactive substances to improve chemical stability [1,2]. According to the different gel matrices, emulsion gels can be divided into protein-based emulsion gels, polysaccharide-based emulsion gels and mixture-based emulsion gels. Protein-based emulsion gels are generally prepared through two-stage processes: first, the preparation of protein emulsions, and then, the gelation of the emulsions induced by heat, acid, salt or enzymes [3]. There are many factors affecting the textural properties of protein-based emulsion gels, and the preparation method of protein emulsions is one of them. In our previous study, we found that compared with high-pressure homogenization, the peanut protein emulsion prepared by the ultrasonic method had improved strength and microstructure after gelation [4]. Additionally, Hu et al. [5] successfully prepared a low oil emulsion gel stabilized by defatted Antarctic krill protein using one-step ultrasound. When whey protein [6] and soy protein [7] solutions were pretreated by ultrasound, followed by preparation of emulsion gels, better texture properties were obtained. These studies have indicated that ultrasonic technology has excellent application potential in the preparation of protein-based emulsion gels.

In addition to the preparation method of protein emulsions, protein composition significantly affected the physicochemical properties of emulsion gels. For example, increasing the soy glycinin content progressively increased the gel stiffness, but significantly decreased the water-holding capacity (WHC). Confocal laser scanning microscopic observations showed that increasing glycinin content led to the formation of emulsion gel network with a more inhomogenous and porous microstructure [8]. Protein composition had a major influence on gel strength, with the strongest emulsion gels being formed at an optimized protein composition (0.5 wt% whey protein and 1.5 wt% lactoferrin) [9]. At fixed total protein content, higher denatured whey protein contents contributed to gels with higher mechanical properties, e.g., fracture stress, Young’s modulus and storage modulus (G′) [10]. Emulsion gels with whey protein isolate at the interface had the highest gel strength (fracture stress and storage modulus), followed by the gels with sodium caseinate, soy protein isolate (SPI), lactoferrin at the interface [11].

Peanut proteins are another important plant protein in addition to soybean proteins, but compared with soybean proteins, the functional properties of peanut proteins are far from being explored, which has seriously limited the applications of peanut proteins in the food industry. Peanut proteins contain two major components: arachin and conarachin. The ratio of arachin to conarachin varies from 0.80 to 1.68 depending on the cultivar of peanuts [12]. Arachin contains two molecular species of arachin, I and II, which possess the same subunit compositions but various molecular weights, 180,000 and 350,000, respectively [13]. Conarachin contains conarachin I and conarachin II, which have different subunit compositions and molecular weights [14]. Monteiro and Prakash [15] compared the functional properties of total protein, arachin, conarachin I, and conarachin II from peanuts, including solubility, water absorption capacity, fat absorption capacity, emulsifying properties, and foaming properties. Liu, Zhao, and Su [16] reported that conarachin had a better emulsifying activity index, foam capacity and heat-induced gelation properties than arachin, while arachin had a higher denaturation temperature, more compacted tertiary conformation and lower surface hydrophobicity than conarachin. Feng et al. [17] found that conarachin and the acid subunits of arachin were more effectively crosslinked by transglutaminase (TGase) than the base subunits of arachin. After TGase treatment, the solubility of both the arachin- and conarachin-rich fractions decreased, and the thermal *T*_d_ of the conarachin-rich fraction significantly increased. More recently, Sun, Zhang, Zhang, Tian, and Chen [18] pointed out that ultrasound-assisted extraction could change the arachin composition and structure, thereby improving its emulsifying properties. Hu, Amakye, He, Wang, and Ren [19] reported that both microfluidization and TGase could effectively unfold the structures of arachin and conarachin, increase the exposed free sulfhydryl (*SH*) content and surface hydrophobicity, and enhance the emulsion stability. Although some structural and functional properties of peanut protein components have been revealed by the above work, little has been studied regarding the preparation and characterization of arachin/conarachin-stabilized emulsion gels.

In this work, emulsions of peanut and soy proteins, including their components, were prepared by ultrasonic methods, and then, the emulsions were further induced by salt (CaCl_2_), TGase and acid (glucono-δ-lactone, GDL) to form emulsion gels. The texture, WHC, dynamic rheological properties and microstructure of different emulsion gels were investigated and compared. Partial structural characteristics of these proteins were analyzed to explore their effects on the emulsion gel properties. This study will promote the understanding of the physical properties of peanut and soybean protein-based emulsion gels induced by various coagulants.

## 2. Results and Discussion

### 2.1. Emulsion Characteristics

**Figure 1:**
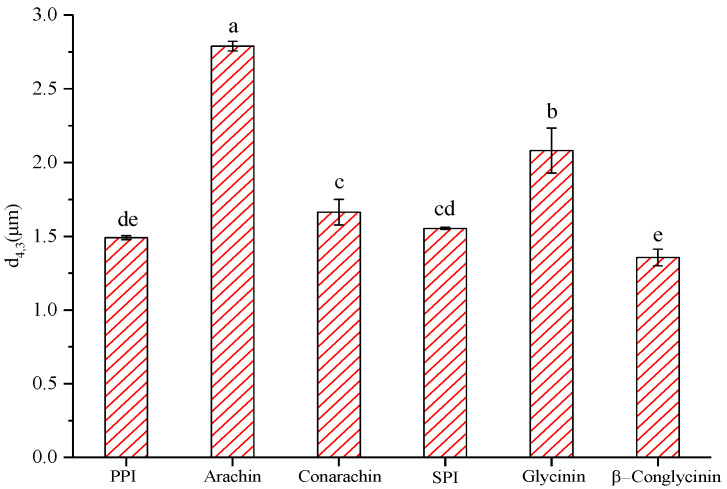
The particle size (*d*_4,3_) of fresh emulsions stabilized by different proteins. The results are expressed as means and standard deviations of three replicates. Bars with different letters indicate significant differences (*p* < 0.05).

**Figure 2:**
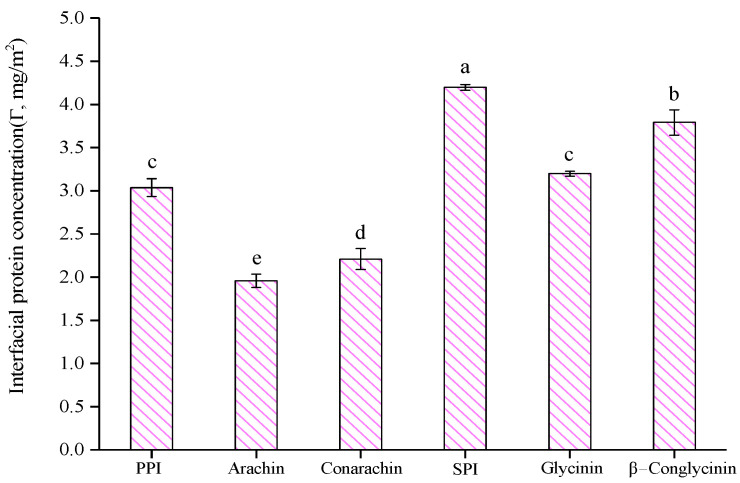
The surface protein concentration (Γ) of fresh emulsions stabilized by different proteins. The results are expressed as means and standard deviations of two replicates. Bars with different letters indicate significant differences (*p* < 0.05).

Based on our previous work [4], the optimal ultrasound conditions (300 W, 20 min) with a constant protein concentration (4%, w/v) and oil fraction (30%, v/v) were selected to prepare emulsions in this study. As shown in Figure 1, among all protein emulsions, the peanut arachin emulsion had the largest particle size (2.79 μm), while the soybean β−conglycinin emulsion had the smallest particle size (1.36 μm), which means that β−conglycinin possessed the strongest emulsifying activity. Regardless of peanut or soybean proteins, the particle size of their low molecular weight fraction-stabilized emulsion was smaller than that of the high molecular weight fraction emulsion. This may be because the low molecular weight protein fractions (such as conarachin and β−conglycinin) can diffuse to the oil-water interface more quickly, preventing the secondary aggregation of oil droplets and resulting in a smaller emulsion particle size. As shown in Figure 2, the Γ of the arachin emulsion was the smallest (1.96 mg/m^2^), whereas that of the SPI emulsion was the largest (4.20 mg/m^2^). Puppo et al. [20] reported a Γ value of 3.03 mg/m^2^ in an SPI emulsion. The difference was probably caused by various emulsifying methods. In our work, ultrasonic treatment likely facilitated the interfacial adsorption of SPI during emulsification. Moreover, Figure 2 shows that the Γ values of SPI and its components were significantly higher than those of peanut proteins isolate (PPI) and its components, which indicated that the interfacial adsorption capacity of soybean proteins was stronger than that of peanut proteins.

### 2.2. Effect of CaCl_2_ Concentration on Hardness and WHC of PPI and SPI Emulsion Gels

**Figure 3:**
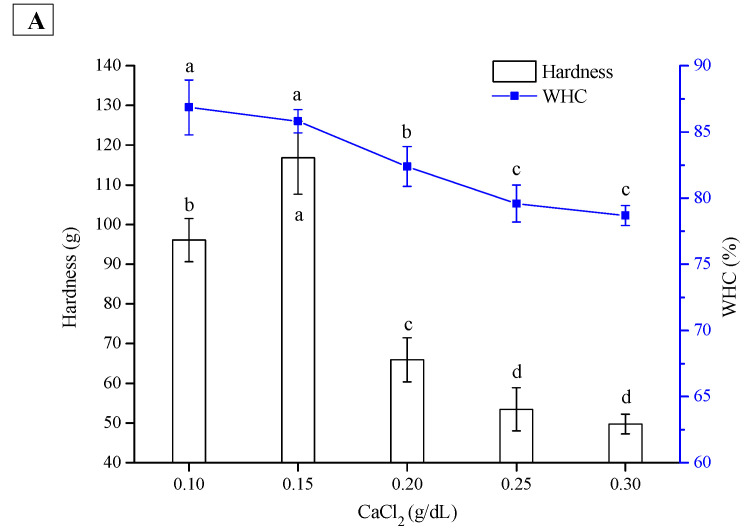

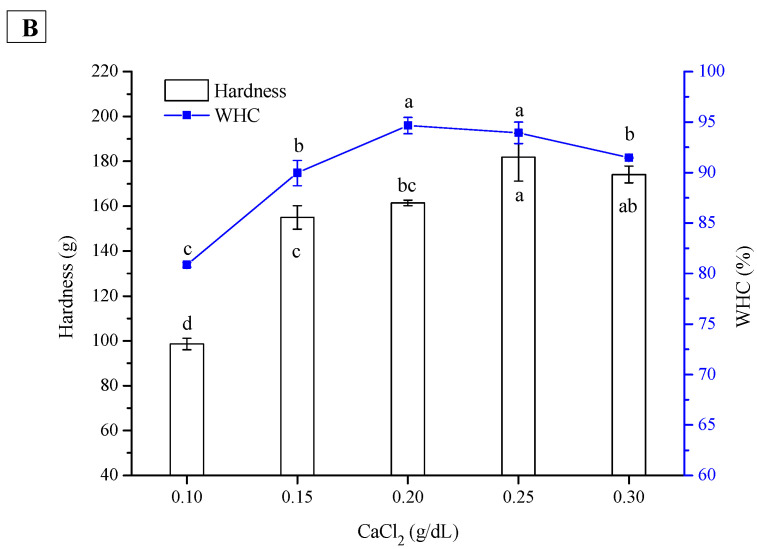
Effect of CaCl_2_ concentration on hardness and WHC of PPI (**A**) and SPI (**B**) emulsion gels. The results are expressed as means and standard deviations of two replicates. Bars with different letters indicate significant differences (*p* < 0.05).

CaCl_2_, as a divalent salt at appropriate concentrations, can not only screen the repulsive forces between protein molecules but also induce the crosslinking of proteins and thus promote gelation. The amount of salt used to form an emulsion gel is a major factor in determining the gel structures. Figure 3A shows that with increasing CaCl_2_ concentration, the hardness of the PPI emulsion gel increased slightly at the beginning and then decreased sharply, while the WHC was gradually reduced. The optimum CaCl_2_ concentration was 0.15 g/dL. Figure 3B shows that the hardness of the SPI emulsion gel was gradually enhanced with increasing CaCl_2_ until its concentration reached 0.25 g/dL, and the gel hardness then decreased slightly. The same trend was observed for the WHC of the SPI emulsion gel. By comparison, the gel hardness and WHC of the PPI emulsions were significantly lower than those of SPI emulsions at the same CaCl_2_ concentrations (except 0.1 g/dL). 

The above results indicated that CaCl_2_ at higher concentrations was not favorable for the gelation of PPI and SPI emulsions. Sok Line, Remondetto, and Subirade [21] found that low Ca^2+^ concentrations induced *β*-lactoglobulin emulsion gels with a fine-stranded structure, while high Ca^2+^ concentrations (such as 68 mM Ca^2+^) reduced the WHC and changed the structure of the emulsion gel from fine-stranded to random aggregates. Wang et al. [22] reported that the storage modulus (G′) of SPI emulsion gels increased gradually with increasing Ca^2+^ concentration (2.5–7.5 mM) but decreased at 10 mM. The decrease in hardness and WHC at higher Ca^2+^ concentrations was probably attributed to random protein aggregation, which led to extremely large protein aggregates forming porous gel structures [21]. It is worth noting that compared with the SPI emulsion gel, the textural deterioration of the PPI emulsion gel was more dramatic at higher Ca^2+^ concentrations, suggesting that the aggregation of PPI might be more sensitive to the Ca^2+^ concentration.

### 2.3. Effect of TGase Concentration on Hardness and WHC of PPI and SPI Emulsion Gels

**Figure 4:**
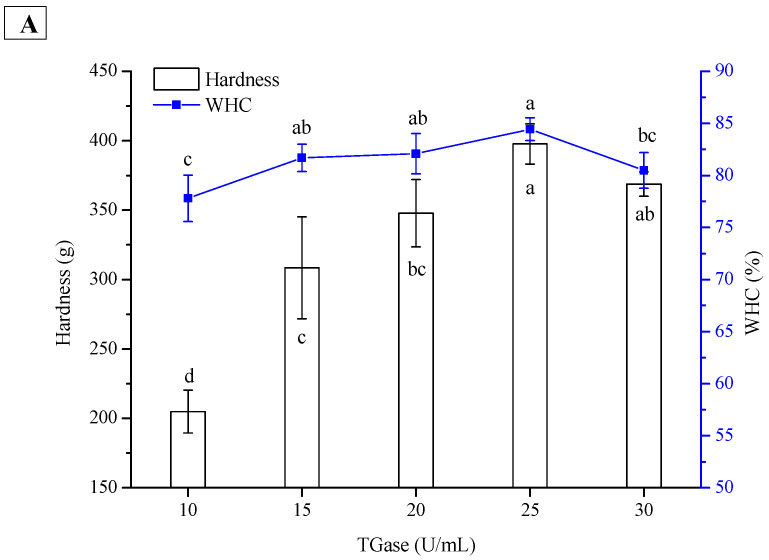

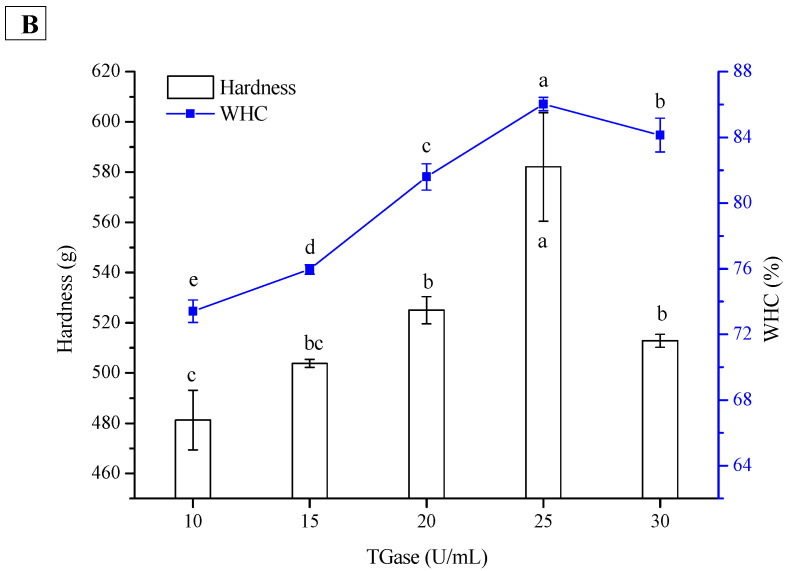
Effect of TGase concentration on hardness and WHC of PPI (**A**) and SPI (**B**) emulsion gels. The results are expressed as means and standard deviations of two replicates. Bars with different letters indicate significant differences (*p* < 0.05).

TGase can catalyze the crosslinking of lysine and glutamine residues on protein molecules, whose strength far exceeds that of hydrophobic interactions and hydrogen bonds [23]. Therefore, this enzyme is widely used in the preparation of cold-set protein gels or emulsion gels. As shown in Figure 4, the hardness and WHC of the PPI and SPI emulsion gels increased with increasing TGase concentration (from 10 to 25 U/mL), suggesting that a more elastic network was developed at higher TGase concentrations. At an appropriate enzyme concentration (25 U/mL), both the hardness and WHC reached the highest values. However, excessive crosslinking between the protein molecules might result in the expulsion of water from the emulsion gels and subsequent textural deterioration. At the same enzyme concentration, the hardness of the PPI emulsion gel was significantly lower than that of the SPI emulsion gel, whereas their WHCs remained similar (Figure 4). In a recent study, Alavi, Emam-Djomeh, Salami, and Mohammadian [24] reported that the hardness of egg white protein gels and emulsion gels increased considerably when TGase increased from 0 to 20 U/g. A higher enzyme concentration was not used in their work.

### 2.4. Effect of GDL Concentration on Hardness and WHC of PPI and SPI Emulsion Gels

**Figure 5:**
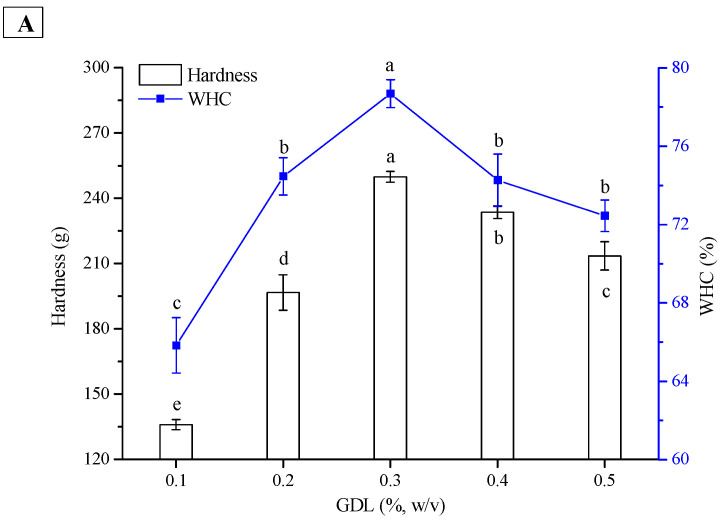

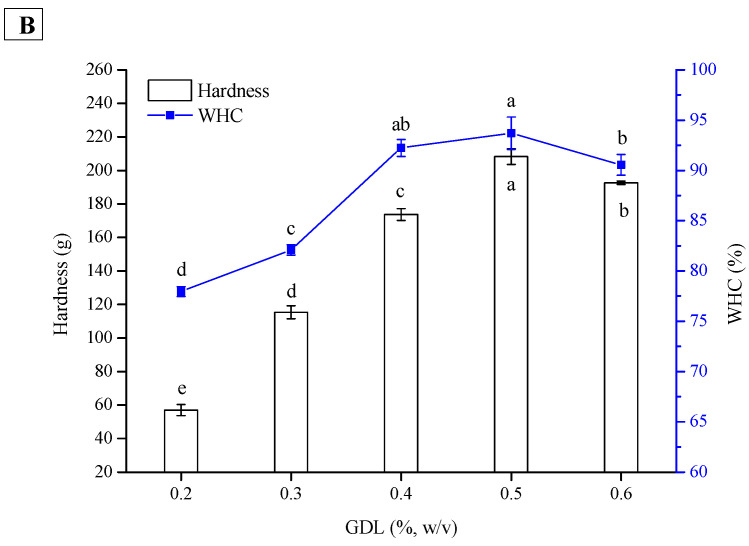
Effect of GDL concentration on hardness and WHC of PPI (**A**) and SPI (**B**) emulsion gels. The results are expressed as means and standard deviations of two replicates. Bars with different letters indicate significant differences (*p* < 0.05).

The mechanism of GDL-induced protein gelation is that acidification decreases the pH and neutralizes the surface charges of protein molecules, and a protein network is then formed by hydrophobic interactions and other intermolecular forces [25]. Figure 5 shows that the hardness and WHC of the PPI and SPI emulsion gels first increased and later decreased as a function of GDL concentration. For the PPI emulsion gel (Figure 5A), both hardness and WHC were the highest at a GDL concentration of 0.3% (w/v). However, for the SPI emulsion gel (Figure 5B), they were the highest at a GDL concentration of 0.5% (w/v). The hardness of the PPI emulsion gel was higher than that of the SPI emulsion gel at the respective optimum concentration, while the WHC of the former was significantly lower. It was reported that soymilk gels induced by GDL were usually in the pH range of 5.0–5.5 [26]. Ringgenberg, Alexander, and Corredig [25] found that at a pH of approximately 5.7, the charge on the soymilk particles was sufficiently diminished to allow aggregation. Therefore, excessive GDL would result in a lower pH in the PPI and SPI emulsions, which was not suitable for gel formation. Moreover, a good linear relationship was observed between the hardness and WHC of the acid-induced emulsion gels (for peanut emulsion gel, R^2^ = 0.907; for soybean emulsion gel, R^2^ = 0.964), suggesting that gel strength played an important role in the water holding of the gels.

### 2.5. Textural Comparison of Protein-Stabilized Emulsion Gels Induced by Various Coagulants and Protein Structural Analysis

**Figure 6:**
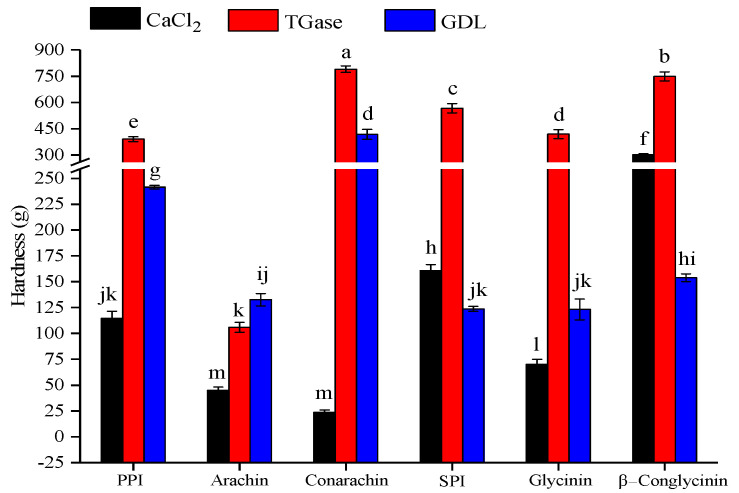
Textural comparison of different protein-stabilized emulsion gels induced by various coagulants. The results are expressed as means and standard deviations of three replicates. Bars with different letters indicate significant differences (*p* < 0.05).

Emulsion gels were prepared using six proteins, including PPI and its major components (arachin and conarachin) and SPI and its major components (glycinin and β−conglycinin), at a constant total protein concentration (4%, w/v) and oil fraction (30%, v/v). The optimal coagulant concentrations were selected based on the above results. As shown in Figure 6, compared to CaCl_2_ and GDL, the hardness of the gels formed by TGase was the highest (except arachin emulsion gel). Tang, Chen, and Foegeding [27] reported that the application of TGase exhibited a much higher potential to form SPI-stabilized emulsion gels with higher mechanical strength than that of the other two coagulants (CaCl_2_ and GDL), which was consistent with our results. As listed in Table 1, since the lysine content of arachin was markedly lower than that of other proteins, it meant that the crosslinking of lysine and glutamine residues on arachin molecules might not be sufficient. On the other hand, although the lysine content of β−conglycinin and SPI was significantly higher than that of conarachin, the gel strength of the latter was higher, which suggested that there were other intermolecular forces other than the isopeptide covalent bonds in the TGase-induced emulsion gels.

**Figure 7:**
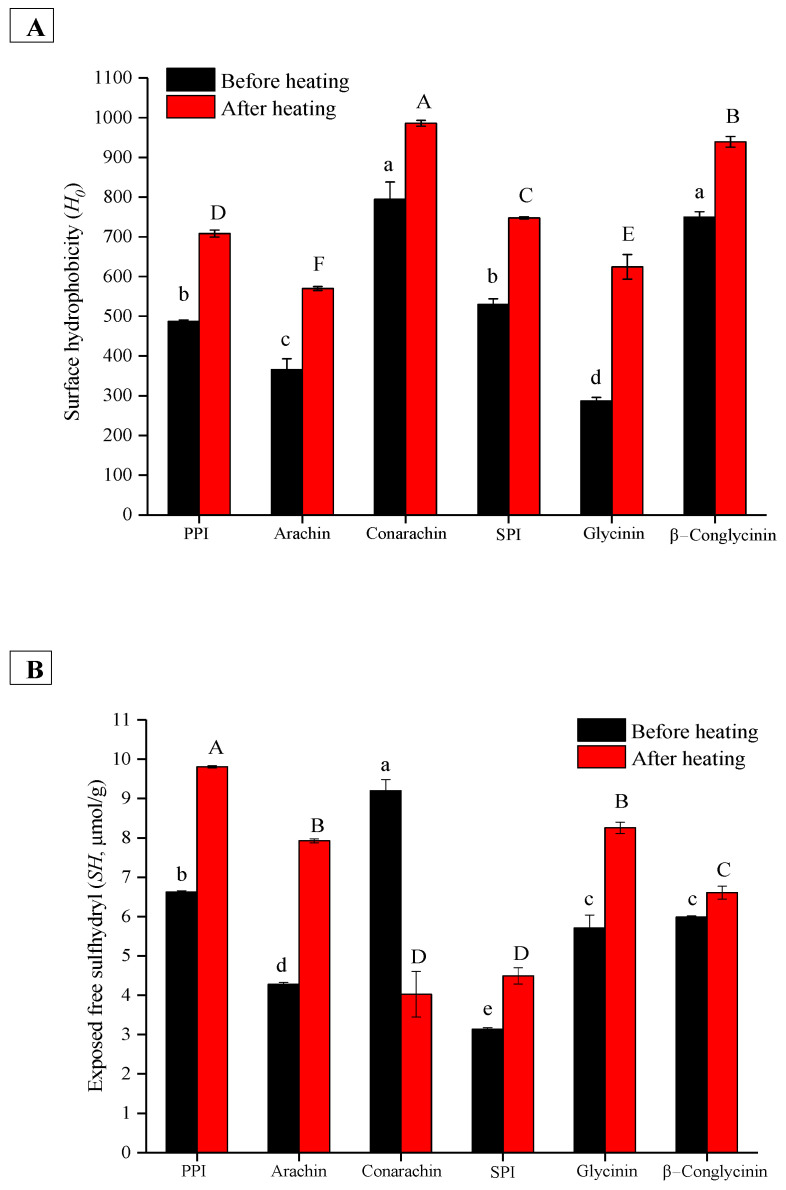
Surface hydrophobicity (**A**) and exposed free sulfhydryl contents (**B**) of different protein samples before and after heating treatment (80 °C for 30 min). The results are expressed as means and standard deviations of two replicates. Bars with different letters indicate significant differences (*p* < 0.05).

Figure 7A displays the surface hydrophobicity (*H*_0_) values of all protein samples before and after heating treatment. Before heating, the *H*_0_ values of conarachin and β−conglycinin were significantly higher than those of other proteins. After heating, conarachin possessed the highest *H*_0_. The results implied that stronger hydrophobic interactions among conarachin molecules could occur during gel preparation. Figure 7B shows the exposed free sulfhydryl (*SH*) contents of all protein samples before and after heating treatment. Before heating, conarachin had the highest exposed SH content (Table 1 shows the cysteine content of conarachin was the highest as well). After heating, the exposed SH content of all samples except conarachin was increased. The significant decrease in the exposed *SH* content of conarachin was probably attributed to the formation of disulfide bonds upon heating treatment. Therefore, it can be concluded that several key intermolecular forces, such as isopeptide bonds, hydrophobic interactions and disulfide bonds, together contribute to the texture of the TGase-induced emulsion gels.

By comparison of the CaCl_2_-induced emulsion gels, the hardness of the β−conglycinin gel was the highest, whereas that of the conarachin gel was the lowest. The results suggested that hydrophobic interactions and disulfide bonds were unlikely to be the key gelation forces when CaCl_2_ was used as the coagulant. Generally, the combination of Ca^2+^ and protein molecules is regarded as H^+^/Ca^2+^ exchange, which neutralizes electrostatic repulsion and forms salt bridges, thus allowing protein molecules to form a network [28]. From the results, it was speculated that salt bridges were not easily formed among conarachin molecules. Although the contents of dicarboxylic amino acids (glutamic acid and aspartic acid) in β−conglycinin and conarachin are similar (Table 1), the exposure of these amino acids, which can provide more protons, to the protein surface may be different, thus affecting H^+^/Ca^2+^ exchanges and the formation of salt bridges.

Among the emulsions using GDL as a coagulant, conarachin emulsions showed the best gelation properties. This can be explained by the fact that when the hydrogen ions released by GDL led to a decrease in the net charge of proteins, conarachin molecules could form a good network structure through strong hydrophobic interactions and the formation of more disulfide bonds. In terms of PPI and its components, the hardness of GDL-induced emulsion gels was significantly higher than that of CaCl_2_-induced emulsion gels. The microstructure and gelation kinetics of CaCl_2_-induced emulsion gels from different proteins were further compared.

### 2.6. Microstructure of Different Protein-Stabilized Emulsion Gels Induced by GDL

**Figure 8:**
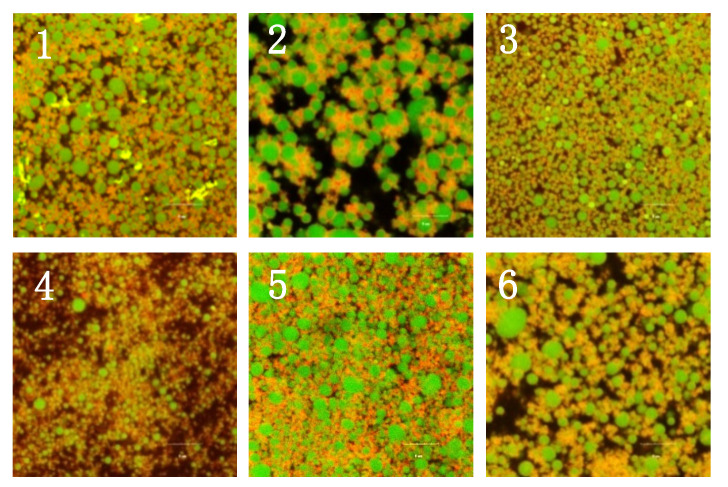
CLSM micrographs of emulsion gel samples induced by GDL, the bars indicate 5 μm in length, oil droplets (green), protein networks (red). 1, PPI; 2, Arachin; 3, Conarachin; 4, SPI; 5, Glycinin; 6, β−Conglycinin.

Generally, the structure of a protein-based emulsion gel is regarded as a composite network made up of a combination of crosslinked protein molecules and partially aggregated droplets [1]. As shown in Figure 8, the spherical oil droplets with green fluorescence were surrounded by a protein network with red fluorescence. There were significant microstructural differences among these samples. Basically, the emulsion gels stabilized by conarachin and PPI showed more homogeneous and compact structures, whereas the arachin emulsion gel exhibited an obviously porous structure and a discontinuous protein network. This result indicated that the interactions among arachin molecules were rather weaker when GDL was used as a coagulant, which may be due to their lower surface hydrophobicity and exposed free sulfhydryl content. Moreover, the particle size of the original arachin emulsion was the largest (Figure 1), and its interfacial protein concentration was the lowest (Figure 2). At the same oil content, the larger the size of the oil droplets, the smaller the number. Since the protein-coated oil droplets could act as active anchors to strengthen the gel texture [1], the decrease in the number of active anchors in the arachin emulsion gel might also be responsible for its worse network. Although the protein network of the glycinin emulsion gel seemed to be dense, it had many large oil droplets, which might weaken the structural strength of the emulsion gel.

### 2.7. Microstructure of Different Protein-Stabilized Emulsion Gels Induced by GDL

**Figure 9:**
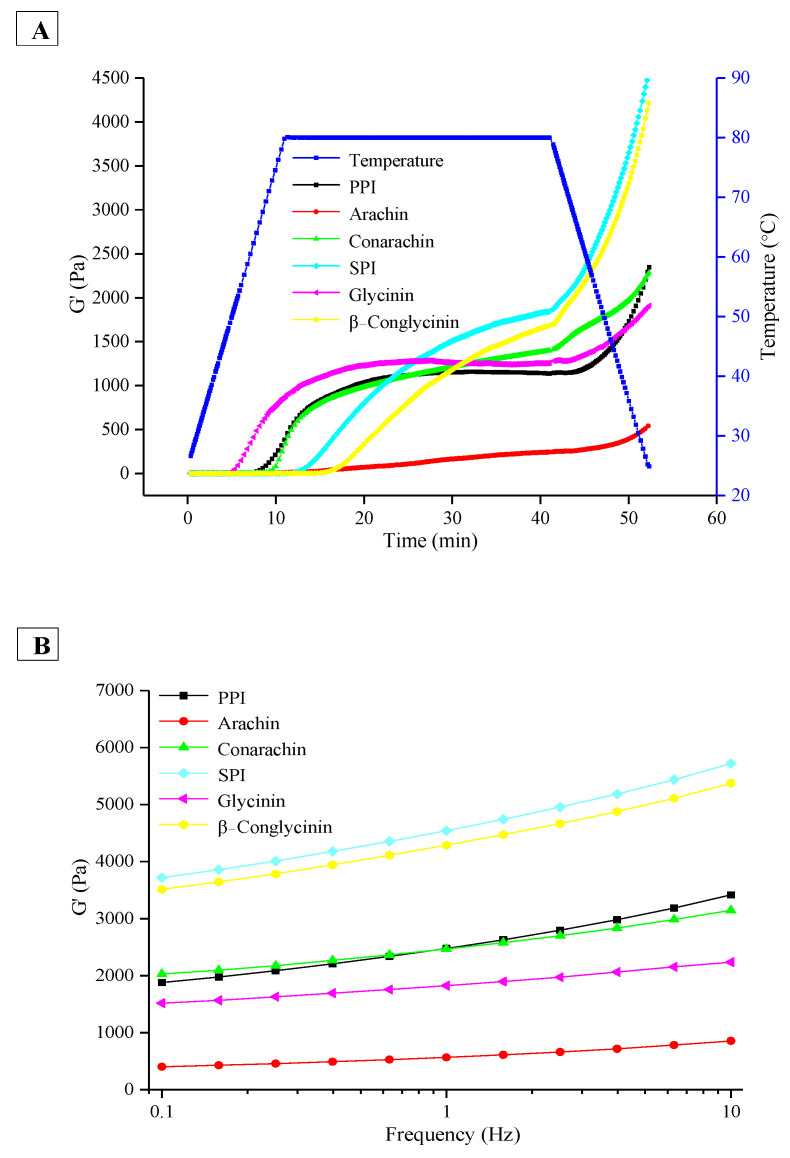
(**A**) Evolution of storage modulus (G′) of emulsion gel samples induced by GDL during heating and cooling cycle. (**B**) Frequency dependence of G′ the correspondingly formed emulsion gels after cooling.

The gelation kinetics of different protein-stabilized emulsions induced by GDL were evaluated. As shown in Figure 9A, during incubation, the storage modulus (G′) of all the emulsions increased sharply except for the arachin-stabilized emulsion. G′ is considered the best indicator of gel structure formation and consolidation [29]. The lower G′ value of the arachin-stabilized emulsion was consistent with its coarse and porous structure. In the initial stage of heating, the G′ value of the glycinin emulsion increased more rapidly than those of other samples. However, after half an hour, the G′ values of SPI and β−conglycinin emulsion gels became higher, which increased more dramatically in the subsequent cooling stage (Figure 9A). Tang, Luo, Liu, and Chen [8] pointed out that G′ progressively increased as the glycinin content increased from 0 to 100% when soy protein emulsion gels were induced by TGase at 37 °C for 6 h. The inconsistent results were probably attributed to the fact that the coagulant and incubation procedure used were different. It is important to note that although the G′ values of conarachin and PPI were much lower than those of the SPI and β−conglycinin emulsion gels according to dynamic rheological analysis, the hardness of the former was remarkably higher after storage overnight at 4 °C (Figure 6). The results imply that the enhancement of intermolecular interactions by storage in conarachin and PPI emulsion gels was higher.

Frequency sweep experiments on the corresponding emulsion gels were further carried out, which could reflect the interactive force nature in the formed emulsion gels. As shown in Figure 9B, the G′ values of arachin and glycinin emulsion gels did not exhibit frequency dependency in the range of 0.1–10 Hz, indicating that these emulsion gels are mainly formed through covalent “chemical crosslinks” [30]. However, in the case of SPI, PPI, β−conglycinin and conarachin emulsion gels, their G′ values gradually increased with increasing frequency, indicating that these emulsion gels are mainly formed through noncovalent “physical crosslinks”, which are breakable or deformable [31]. Wang et al. [22] and Tang, Yang, Liu, and Chen [8] reported similar results when using various coagulants.

## 3. Conclusions

The various coagulant concentrations were optimized for peanut and soy protein-stabilized emulsion gels. The soy protein gel generally had better WHC than the peanut protein gel. Among the six proteins (PPI, arachin, conarachin, SPI, glycinin and β−conglycinin), when CaCl_2_ was used as a coagulant, the hardness of the emulsion gel from conarachin was the lowest. However, when TGase and GDL were used as coagulants, the conarachin emulsion gel had the strongest texture. The GDL-induced conarachin emulsion gel showed more homogeneous and compact structures. Protein structural analysis showed that conarachin had the highest surface hydrophobicity and exposed free sulfhydryl content, which should be responsible for the better texture of the conarachin-stabilized emulsion gel. This work reveals that conarachin has good application potential in the preparation of emulsion gels using TGase and GDL as coagulants.

## 4. Materials and Methods

### 4.1. Materials

Peanut seeds (4.53% water, 49.81% oil and 25.07% protein), defatted soybean meal and soybean oil were purchased from the local market. PPI (protein content: 80.15 ± 1.69%) was prepared according to the procedure of alkali-soluble and acid precipitation in our lab [4]. The oil content of the peanut seeds was determined by the Soxhlet extraction method (AOCS Official Method, Ba 3-38, 1998). The protein contents of the peanut seeds and PPI were determined by the Kjeldahl method (N × 5.46) (AOCS Official Method, Ba 4a-38, 1998). 8-Anilinonaphthalene-sulphonic acid (ANS), 5,5’-dithiobis (2-nitrobenzoic acid) (DTNB), Nile red and Fluorescein isothiocyanate (FITC) were purchased from Yuanye Bio-Technology Co., Ltd. (Shanghai, China). GDL was purchased from Macklin Biochemical Co., Ltd. (Shanghai, China). The microbial TGase used in this study was Baibang TGase (a solid powder mixed with maltodextrin, with an enzyme activity of 242 U/g) donated by Qingrui Food Technology Co., Ltd. (Shanghai, China). All other reagents were of analytical grade.

### 4.2. Extraction of Arachin and Conarachin from PPI

PPI was mixed with 3 times phosphate buffer solution (0.3 mol/L, pH 7.5), which was stirred at room temperature for 1 h, followed by centrifugation (4000 r/min, 15 min) at room temperature with a centrifuge (GL-20G, Anting Instrument Co, Ltd., Shanghai, China) to discard the insoluble matter. The supernatant was treated in two ways to obtain arachin and conarachin. (1) The supernatant was placed overnight at 2 °C and then centrifuged (8000 r/min, 20 min) at 2 °C. The precipitated arachin was freeze-dried and stored in a desiccator. (2) Ammonium sulfate was added to the supernatant until its saturation reached 60%. The solution was stirred evenly and then centrifuged (8000 r/min, 20 min). Ammonium sulfate was added to the supernatant until its saturation reached 85%. The solution was stirred and then centrifuged as described above. The precipitate was dialyzed to remove ammonium sulfate, followed by freeze drying to obtain conarachin. The protein contents of arachin and conarachin were 84.45 ± 0.51% and 89.85 ± 0.98%, respectively.

### 4.3. Extraction of Soy Protein Isolates (SPI), Glycinin and β−Conglycinin from Defatted Soybean Meal

SPI was extracted according to the alkali-soluble and acid precipitation procedure. Soy glycinin and β−conglycinin were prepared according to the process reported by Nagano, Hirotsuka, and Mori [32]. The specific steps were as follows.

(1) Defatted soybean meal was mixed with 10 times deionized water, and the pH value of the dispersions was adjusted to 8.0 with 2 M NaOH. After stirring for 1 h at room temperature, the suspension was shaken in a water bath shaker (SHZ-88, Jiangsu Instrument Co., Ltd., Jingyi, China) at 50 °C for 30 min. After centrifugation (4000 r/min, 15 min), the precipitate was discarded, and the pH of the supernatant was then adjusted to 4.5 with 2 M HCl to precipitate the soy proteins. The precipitate was then washed with deionized water twice to remove most of HCl. The precipitated proteins were freeze-dried as SPI (protein content: 83.60 ± 1.0%).

(2) Sodium bisulfite was added to the above supernatant to achieve a final concentration (0.98 g/L), and its pH was adjusted to 6.4 with 2 M HCl. The protein solution was then placed overnight at 4 °C, followed by centrifugation (4 °C, 8000 r/min, 20 min). The obtained precipitate was freeze-dried as glycinin (87.94 ± 0.66%). Next, sodium chloride was added to the obtained supernatant to achieve a final concentration (0.25 mol/L), and its pH was adjusted to 5.0 with 2 M HCl. The protein solution was then centrifuged at 8000 r/min for 20 min. The precipitate was discarded. Twice the volume of ice water was added to the supernatant, and the pH was adjusted to 4.8, followed by centrifugation (4 °C, 8000 r/min, 20 min). The obtained precipitate was freeze-dried as β−conglycinin (82.37 ± 0.8%).

### 4.4. Preparation of Different Protein-Stabilized Emulsions by Ultrasonic Method

First, protein suspensions (4%, w/v) were prepared by dispersing the protein samples in distilled water into a 200-mL beaker, which was gently stirred at 25 °C until they were sufficiently dissolved. The pH of the dispersions was adjusted to 7.0 with 2 M NaOH. Then, soybean oil (30%, v/v) was added to the protein suspensions. The mixture was processed at 10,000 rpm for 2 min by a high-shear probe homogenizer (Model FA25, Fluko Equipment Shanghai Co., Ltd., Shanghai, China). The obtained coarse emulsions were further treated by an ultrasonic processor (Scientz-IID, NingBo Scientz Biotechnology Co., Ltd., Ningbo, China) with an 80–170 μm of amplitude to prepare the final emulsions. The titanium probe used had a 0.636-cm diameter. The samples were sonicated (20 min, pulse duration of 3 s and off time of 2 s) at 300 W. The sample temperature was kept below 30 °C in an ice-water bath.

### 4.5. Emulsion Characterization 

The particle size of the emulsion samples was measured according to our previous work [4]. The surface protein concentration of the emulsion samples was measured according to the method of Zhang and Lu [33].

### 4.6. Preparation of Emulsion Gels 

All emulsion samples were prepared at a constant total protein concentration (4%, w/v) and oil fraction (30%, v/v). The emulsion gels were prepared using CaCl_2_, TGase and GDL were used as the coagulants in 50-mL beakers.

(1) CaCl_2_-induced gels. CaCl_2_ was added to the emulsion sample (20 mL) to achieve final concentrations of 0.1, 0.15, 0.2, 0.25, and 0.3 g/dL. The mixture was gently stirred and then heated for 30 min in an 80 °C water bath to form a gel. The gel was then cooled immediately in cold water and stored overnight at 4 °C.

(2) TGase-induced gels. TGase was added to the emulsion sample (20 mL) to achieve final concentrations of 10, 15, 20, 25 and 30 U/mL. The mixture was stirred for 3 min at room temperature, followed by incubation in a 45 °C water bath for 6 h to form gels. Then, the gels were cooled immediately and stored overnight at 4 °C.

(3) GDL-induced gels. GDL was added to the emulsion sample (20 mL) to achieve a final concentration (0.1%, 0.2%, 0.3%, 0.4%, 0.5% and 0.6% (w/v)). The mixture was gently stirred and then heated for 30 min in an 85 °C water bath to form a gel. The gel was then cooled immediately in cold water and stored overnight at 4 °C. The texture and WHC of the emulsion gels were analyzed to establish the concentrations of coagulants.

### 4.7. Emulsion Gel Characterization 

#### 4.7.1. Dynamic Viscoelastic Measurement

Dynamic viscoelastic measurements of emulsion gels were determined at a 1.0% strain according to our previous work [4]. The storage modulus (G′) and loss modulus (G″) were recorded during heating and cooling cycles at a fixed frequency of 1.0 Hz. Then, the experiment was oscillated at 25 °C with a frequency from 0.1 to 10.0 Hz. G′ and G″ were recorded.

#### 4.7.2. Emulsion Gel Textural Analysis

The texture of the emulsion gels was analyzed by a texture analyzer (TA-TX2i, Stable Micro System Ltd., Godalming, England) with a cylinder probe (P/0.5) according to our previous work [4].

#### 4.7.3. Emulsion Gel Water-Holding Capacity (WHC)

The emulsion gel samples (5.0–6.0 g each) were transferred to 10-mL centrifuge tubes and centrifuged (4 °C, 8000 r/min, 20 min). The water on top of the tubes was carefully removed using filter paper. The tubes containing the emulsion gels before and after centrifugation were weighed. The WHC was calculated using Equation (1):

(1) $${\mathrm{WHC}\;(\%)=(W}_{1}-W)/{(W}_{2}-W)\times 100\%$$

where W is the weight of the centrifuge tube; W_1_ is the total weight of the centrifuge tube and emulsion gel after centrifugation; and W_2_ is the total weight of the centrifuge tube and emulsion gel before centrifugation.

#### 4.7.4. Confocal Laser Scanning Microscope (CLSM)

A confocal laser scanning microscope (FV3000, Olympus, Tokyo, Japan) equipped with a 100× objective lens (oil immersion) was applied to observe the microstructure of GDL-induced emulsion gels. The assay procedure was performed according to our previous work [4].

### 4.8. Protein Structural Properties 

#### 4.8.1. Surface Hydrophobicity 

The surface hydrophobicity (*H*_0_) of protein samples was determined using ANS as the fluorescence probe according to our previous work [4].

#### 4.8.2. Exposed Free SH Content 

The exposed free *SH* content of the protein samples was determined using the 5,5’-dithiobis (2-nitrobenzoic acid) (DTNB) reagent according to the method of Zhang, Yan, Jiang, and Ding [34] with modifications. Ellman’s reagent was prepared by dissolving 40 mg of DTNB reagent in 10 mL of tris-glycine buffer (pH 8.0) containing 0.086 M Tris, 0.09 M glycine and 4 mM EDTA. Protein samples (30 mg) were solubilized in 10 mL of Tris-glycine buffer, followed by centrifugation at 5000 r/min for 15 min. The protein content of the supernatant was determined by the method of Lowry, Rosebrough, Farr, and Randall [35]. Then, 50 μL of Ellman’s reagent was added to 3 mL of supernatant. After incubation at room temperature for 5 min, the absorbance of the suspension was read at 412 nm. The buffer was used as a control blank. The *SH* content was calculated using Equation (2):

(2) $$SH\;(\mu \mathrm{mol}/g)=\frac{73.53\;A}{C}$$

where A is the absorbance at 412 nm and C is the protein concentration of the supernatant (mg/gL).

#### 4.8.3. Amino Acid Analysis

Seventeen amino acids (except for tryptophan) were analyzed using an automated amino acid analyzer (S433D, Sykam, Germany). After hydrolyzing the protein samples at 110 °C for 24 h with 6 M HCl, RP-HPLC analysis with online derivation was carried out. 

### 4.9. Statistical Analysis

The reported values are the means for at least two replicates. The significant difference (*p* < 0.05) between various samples was analyzed through ANOVA by Duncan’s multiple range tests.

## Figures and Tables

**Table 1:** Amino acid composition of peanut and soy proteins including their components (Grams per 100 g of Protein). The results are expressed as means and standard deviations of two replicates. Bars with different letters in each line indicate significant differences (*p* < 0.05).

	PPI	Arachin	Conarachin	SPI	Glycinin	β−Conglycinin
Asp	12.55 ± 0.003 ^b^	13.12 ± 0.39 ^a^	12.56 ± 0.33 ^b^	12.36 ± 0.82 ^bc^	12.23 ± 0.13 ^cd^	12.10 ± 0.72 ^d^
Thr	2.49 ± 0.04 ^d^	2.18 ± 0.01 ^e^	2.19 ± 0.002 ^e^	3.25 ± 0.09 ^b^	3.58 ± 0.15 ^a^	2.8 ± 0.02 ^c^
Ser	4.77 ± 0.01 ^c^	4.71 ± 0.09 ^c^	4.68 ± 0.02 ^c^	5.01 ± 0.01 ^b^	4.99 ± 0.13 ^b^	5.23 ± 0.001 ^a^
Glu	21.61 ± 0.10 ^d^	22.64 ± 0.06 ^c^	23.48 ± 0.09 ^b^	21.81 ± 0.09 ^cd^	22.59 ± 0.55 ^c^	24.53 ± 0.03 ^a^
Gly	4.53 ± 0.04 ^a^	4.12 ± 0.01 ^c^	4.212 ± 0.01 ^bc^	4.12 ± 0.01 ^c^	4.34 ± 0.09 ^b^	3.31 ± 0.08 ^d^
Ala	4.02 ± 0.02 ^a^	4.01 ± 0.01 ^a^	3.14 ± 0.003 ^c^	3.66 ± 0.01 ^b^	3.70 ± 0.08 ^b^	3.26 ± 0.09 ^c^
Cys	0.50 ± 0.001 ^ab^	0.45 ± 0.05 ^b^	0.80 ± 0.01 ^a^	0.56 ± 0.11 ^ab^	0.57 ± 0.17 ^ab^	0.45 ± 0.05 ^b^
Val	4.53 ± 0.05 ^bc^	4.34 ± 0.01 ^c^	4.33 ± 0.04 ^c^	4.69 ± 0.03 ^b^	4.97 ± 0.14 ^a^	4.10 ± 0.02 ^d^
Met	1.27 ± 0.002 ^b^	0.77 ± 0.004 ^d^	2.47 ± 0.02 ^a^	1.14 ± 0.03 ^c^	1.2 ± 0.02 ^b^	0.81 ± 0.04 ^d^
Ile	3.74 ± 0.03 ^c^	3.57 ± 0.02 ^d^	3.81 ± 0.02 ^c^	4.83 ± 0.04 ^a^	4.53 ± 0.09 ^b^	4.75 ± 0.04 ^c^
Leu	6.89 ± 0.03 ^c^	6.86 ± 0.04 ^c^	5.95 ± 0.01 ^d^	7.40 ± 0.06 ^b^	7.47 ± 0.03 ^b^	7.68 ± 0.05 ^a^
Tyr	4.45 ± 0.001 ^b^	5.08 ± 0.01 ^a^	1.92 ± 0.01 ^e^	3.77 ± 0.01 ^c^	3.70 ± 0.06 ^c^	3.50 ± 0.002 ^d^
Phe	5.44 ± 0.01 ^b^	5.88 ± 0.02 ^a^	4.38 ± 0.02 ^d^	5.43 ± 0.03 ^b^	5.19 ± 0.10 ^c^	5.82 ± 0.03 ^a^
His	3.00 ± 0.01 ^b^	2.91 ± 0.02 ^b^	3.21 ± 0.03 ^a^	3.31 ± 0.0001 ^a^	2.96 ± 0.10 ^b^	2.91 ± 0.03 ^b^
Lys	3.32 ± 0.06 ^d^	2.29 ± 0.01 ^e^	5.02 ± 0.02 ^c^	5.98 ± 0.05 ^b^	5.00 ± 0.12 ^c^	6.26 ± 0.05 ^a^
Arg	12.57 ± 0.03 ^c^	13.05 ± 0.05 ^b^	13.98 ± 0.04 ^a^	7.71 ± 0.03 ^e^	7.60 ± 0.06 ^e^	8.09 ± 0.04 ^d^
Pro	4.34 ± 0.18 ^bc^	4.00 ± 0.32 ^c^	3.85 ± 0.021 ^c^	4.97 ± 0.022 ^ab^	5.33 ± 0.08 ^a^	4.31 ± 0.08 ^bc^

## Data Availability

The data presented in this study are available on request.
